# Advancements in Adenine Nucleotides Extraction and Quantification from a Single Drop of Human Blood

**DOI:** 10.3390/molecules29235630

**Published:** 2024-11-28

**Authors:** Ivana Popović, Lucija Dončević, Renata Biba, Karla Košpić, Maja Barbalić, Mija Marinković, Mario Cindrić

**Affiliations:** 1 Doctoral Study of Biophysics, Faculty of Science, University of Split, 21000 Split, Croatia; ipopovic@pmfst.hr; 2 Faculty of Science, University of Split, 21000 Split, Croatiammarinkov@pmfst.hr (M.M.); 3 Division of Molecular Medicine, Ruđer Bošković Institute, 10000 Zagreb, Croatiarenata.biba@irb.hr (R.B.); 4 Faculty of Biotechnology and Drug Development, University of Rijeka, 51000 Rijeka, Croatia; karla.kospic@biotech.uniri.hr

**Keywords:** adenine nucleotides, adenylate energy charge, capillary blood samples, micro-solid phase extraction, activated carbon purification

## Abstract

Adenine nucleotides (ANs)—adenosine 5′-triphosphate (ATP), adenosine 5′-diphosphate (ADP), and adenosine 5′-monophosphate (AMP)—are essential for energy transfer and the supply of countless processes within cellular metabolism. Their concentrations can be expressed as adenylate energy charge (AEC), a measure of cellular metabolic energy that directly correlates with the homeostasis of the organism. AEC index has broad diagnostic potential, as reduced ATP levels are associated to various conditions, such as inflammatory diseases, metabolic disorders, and cancer. We introduce a novel methodology for rapid isolation, purification, and quantification of ANs from a single drop of capillary blood. Of all the stationary phases tested, activated carbon proved to be the most efficient for the purification of adenine nucleotides, using an automated micro-solid phase extraction (µ-SPE) platform. An optimized µ-SPE method, coupled with RP-HPLC and a run time of 30 min, provides a reliable analytical framework for adenine nucleotide analysis of diverse biological samples. AN concentrations measured in capillary blood samples were 1393.1 µM, 254.8 µM, and 76.9 µM for ATP, ADP, and AMP molecules aligning with values reported in the literature. Overall, this study presents a streamlined and precise approach for analyzing ANs from microliters of blood, offering promising applications in clinical diagnostics.

## 1. Introduction

Adenosine 5′-triphosphate (ATP), adenosine 5′-diphosphate (ADP), and adenosine 5′-monophosphate (AMP), i.e., adenine nucleotides (ANs), are crucial molecules in all living organisms. In humans, they have a pivotal role in energy transfer and storage, but also in numerous physiological processes, such as neurotransmission, mechanosensory transduction, vasodilation, as well as cellular signaling, development, and regeneration [1]. Detection and quantification of ANs in biological samples are crucial for monitoring degradation of these phosphorylated compounds and thus evaluating the energy status of organisms [2]. Adenylate energy charge (AEC), first described by Atkinson [3] in 1968, serves as a fundamental measure for assessing the energy status of a cell. It is defined by the following equation:

(1) $$AEC=\frac{\left([ATP]+\frac{1}{2}[ADP]\right)}{\left([ATP]+[ADP]+[AMP]\right)}$$

AEC can be affected by the catalytic properties of enzymes involved in both catabolic and biosynthetic metabolic pathways, emphasizing its highly regulated nature [4]. Zhang and Vertes [5] reported that in healthy cells, AEC typically falls within the range of 0.80–0.95. Conversely, an AEC value of 0.5 or below indicates cell death, which can be caused by apoptosis, necrosis, or autophagy [6,7]. The determination of AN concentration in human blood and other biological samples (e.g., follicular fluid, seminal plasma) is therefore of immense importance for understanding metabolic or pathological conditions and for monitoring the overall energy status of the human body.

Researchers have found that the energy stored in AN molecules is an indicator of an organism’s overall health and have linked a significant decrease in AEC to pathological conditions and disease. Namely, Coolen et al. [7] and Aragon-Martinez et al. [2] for the first time developed and monitored AN levels and ATP-related compounds, extracted from small volumes of human venous blood (less than 1 mL), to evaluate the energy status of erythrocytes. Domanski et al. [8] and Marlewski et al. [9] indicated higher values of ATP in human red blood cells of patients with chronic renal failure, which can be explained with the accelerated nucleotide synthesis in uremic erythrocytes. Therefore, uremic erythrocytes are classified as hypermetabolic cells, characterized by a 70% increase in AN concentration compared to healthy subjects [10,11]. Zhang et al. [12] found that AN concentrations are higher in tumor cell lines in comparison to the normal cells which indicated abnormal metabolism of nucleotides in tumor cells. Ledderose et al. [13] showed that AN levels are lower in children than in adults because of increased activity of the enzyme responsible for their breakdown. This deficiency reduces the effectiveness of neutrophils and macrophages in immune response, making children more prone to bacterial infections.

The quantitative analysis of adenine nucleotides and the determination of AEC in living organisms have extended their purpose beyond mere organism health monitoring. Previous studies have underscored the significance of AEC levels as a physiological measure of environmental stress and health index. It can be used to assess the ecology of local rivers [14], contaminated forest soils [15], oil polluted seas [16], for monitoring the organismal environmental adaptation [17], assessing the effects of global climate changes [18], and various other ecological niches.

Previous studies [2,7,8,9,13,19], focusing on AN analysis extracted from human blood typically required large volumes (up to 8 mL) of venous blood samples stored in EDTA-containing vacutainer tubes to prevent blood clotting. However, it was discovered that anticoagulants promote faster ATP hydrolysis which can interfere with downstream measurements, leading to unreliable results [19]. The analysis of ANs from small (microliter) amounts of capillary blood would therefore eliminate the need for sample storage and the use of anticoagulants. The most widespread method for blood AN analysis is high-performance liquid chromatography (HPLC) [2,7,8,20,21,22,23,24], which enables simultaneous quantification of all ANs in a single run, unlike other known methods, like bioluminescent ATP assay, which is limited to the measurement of ATP only [25,26]. Furthermore, a study conducted by Yeung et al. [20] reported difficulties while measuring the purine nucleotide concentrations from red blood cells due to interference from biomolecules and various cell metabolites. Since biological samples contain high level of proteins and other metabolites which could hinder the detection and quantification of ANs [20,27], proper sample preparation prior to HPLC analysis is essential. The preparation starts with sample quenching to suppress enzymatic processes which could alter AN concentrations [28]. The most efficient methods include protein precipitation with strong acid, such as perchloric acid (PCA) or trichloroacetic acid (TCA), which halt all phosphatase activity that could dephosphorylate ANs [29,30,31]. A neutralization step that follows includes acid removal via precipitation for PCA [32], while TCA requires further liquid-liquid extraction which can lead to unwanted analyte alterations and lower recovery rates [33,34]. Furthermore, purification during sample preparation represents an essential step for the removal of other polar metabolites which could interfere with the detection and quantification of ANs. In this context, solid phase extraction (SPE), a commonly used technique for the isolation and concentration of analytes, can contribute to increasing sensitivity by reducing the complexity of the sample [35,36]. Moreover, recent advancements in automated micro-solid phase extraction (µ-SPE) procedures can overcome problems associated with manual SPE, like low reproducibility and recovery rates. On top of that, smaller column diameters ensure smaller inlet and outlet void volumes that enable quantitative purification of low analyte volumes, require smaller amounts of solvents, and result in overall higher efficiency of extraction [37]. Previous research reported the SPE approach in the purification of different nucleotides using different stationary phases. Common approaches utilize reverse-phase chromatography, using Strata-X [27,38], or Sep-Pak C18 SPE cartridges [37]. Affinity chromatography using boronate [14] or phenyl-boronate [39] stationary phase was also reported for environmental samples. Moreover, ion exchange was proven successful for nucleotide purification and separation, especially anion exchange chromatography [32,38], which uses a positively charged stationary phase with which the negatively charged nucleotides interact [40]. All of the abovementioned SPE methods need thorough adjustments to provide good results for each sample type, and usually only work as fractionation methods dividing analyzed nucleotides in several fractions for further analysis. This creates a need for a simple protocol which could purify AN samples in one step and in that way decrease the overall analysis time.

In the presented work, we introduce a novel methodological study for the rapid and accurate extraction, identification, and quantification of ANs from human blood. We introduce an optimized automated μ-SPE method using activated carbon as the stationary phase which enables fast and reproducible purification of ANs. The extraction method was subsequently analyzed both qualitatively and quantitatively using an RP-HPLC method with diode array detector (DAD). The RP-HPLC method was validated with respect to linearity range, selectivity, inter-day, and intra-day precision, LOD, LOQ, recovery, and stability. By providing robust methods for purification, identification, and quantification of ANs applicable for a wide range of biological samples, our study contributes to a deeper understanding of ecological, metabolic, and pathological conditions.

## 2. Results and Discussion

### 2.1. Protein Precipitation and Membrane Filtration for Adenine Nucleotides Extraction

The first objective of this research was to successfully extract ANs from the smallest possible amount of blood; that is, one drop (around 50 µL), collected from a finger. Previous studies analyzed ANs and other analytes extracted from more than 120 µL of human venous blood [2,7,13,23,24]. For that purpose, up to 8 mL of venous blood had to be sampled and stored in an anti-coagulant containing vacutainer tubes to prevent clotting. However, studies have shown that storing blood in tubes with anticoagulants after venipuncture accelerates the hydrolysis of ATP to ADP, AMP, and adenosine [19,41]. The novel methodology presented in this work shows the advantage of performing AN extraction and analysis immediately after sampling capillary blood, thereby preventing further ATP hydrolysis. Even though venipuncture (collection of venous blood) represents the standard technique for blood sample acquisition, it requires expertise, larger volumes of blood, and can be challenging in special populations. Capillary sampling therefore represents a faster and easier sample collection technique with significantly less sample volume and manipulation [42].

Blood serum and blood plasma are complex biological mixtures that contain numerous endogenous components, the majority of which account for proteins, followed by various salts and lipids, which can all interfere with the analytes during the analysis. On top of that, proteins, especially in their native form, pose an additional technical problem as they can interfere with the chromatographic support, which impairs the separation performance and can lead to clogging of the column [43]. A rapid and efficient procedure to remove proteins in biological samples is protein precipitation. This process usually involves the addition of an appropriate reagent, often an organic solvent, acids, or salts, to reduce the solubility of proteins, separating it from the solution by centrifugation or filtration to create a particulate-free supernatant or filtrate [44,45,46,47]. Precipitation with PCA is one of the most used deproteinization protocols, as it not only removes most of the proteins present in the sample, but also stabilizes many of the small molecules [48]. The addition of PCA is also the most common approach for nucleotide extraction from biological samples [12,14], but it has also been used successfully in the preparation of samples prior to quantification of a number of small molecules, including glycogen, ATP, cAMP, glutathione, antioxidants, etc. [49].

In this work, a successful extraction of ATP, ADP, and AMP from 50 µL of capillary blood was achieved by adding an equal volume of ice-cold 8% PCA solution to precipitate proteins and inactivate enzymes, mainly phosphatases, which could potentially disrupt AN levels. Since residual acid causes ATP hydrolysis, the sample was neutralized with a sodium carbonate buffer solution, forming an insoluble precipitate of sodium perchlorate. It has already been reported that a certain amount of ATP can be lost by adsorption to perchlorate precipitate [50], which should be considered when interpreting the results. After the neutralization step, the remaining supernatant was visibly turbid. To prevent eventual HPLC instrument clogging, we included an additional separation step based on molecular weight. Namely, filtration using a molecular weight cut-off (MWCO) filter, which was used to retain all the compounds with a molecular weight larger than 10 kDa, including cell debris and remaining proteins, as well as multivalent ions, sugars, and other organic compounds [51]. The concentrated filtered solution contained smaller molecular weight compounds, including ANs.

### 2.2. µ-SPE Purification of Adenine Nucleotides

Small molecules, such as fatty acids, carbohydrates, amino acids, nucleotides, etc., are frequently hindered by the presence of proteins in biological samples, making their analysis difficult without extensive purification procedures. Due to the complexity of biological samples, optimizing the purification procedure is necessary to achieve satisfactory chromatographic separation and subsequent detection of the analytes [52]. To address this need, nine different stationary phases were tested for ANs standard solution purification to find a suitable µ-SPE method for further purification of ANs extracted from biological samples. These stationary phases were selected based on the previous research, and included different separation mechanisms: weak anion exchange (WAX), Cyano, Quaternary methyl ammonium (QMA), QMA in combination with Hydrophilic–lipophilic balanced polymer (HLB), reverse-phase (C18), reverse-phase Strata X, C18 in combination with Strata X, Affi-gel Boronate, and activated carbon [14,20,36,53,54,55,56,57,58]. Among the nine evaluated stationary phases, only the activated carbon as a stationary phase selectively bound and eluted ANs. Consequently, activated carbon was chosen as the stationary phase for the purification of ANs from human blood. Binding of ANs to activated carbon is based on the interaction between the electrons in the aromatic rings of the purine and pyrimidine nucleotide aromatic rings and the free π-electrons of the activated carbon [53,59].

**Figure 1:**
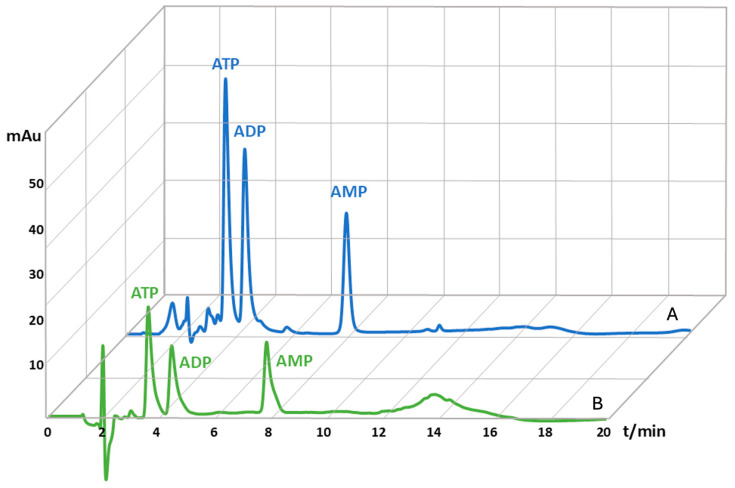
Magnified HPLC chromatograms of adenine nucleotides extracted from human capillary blood, mixed with spike solution in the concentration of 200 µM, prior to µ-SPE purification (A) and adenine nucleotides extracted from human capillary blood, mixed with spike solution in the concentration of 200 µM, after the µ-SPE purification (B). The x-axis represents retention time in minutes, and the y-axis measures a specific signal generated by a detector in milli-absorbance units (mAu).

After the capillary blood extraction, protocol samples were slightly red and turbid, whereas the samples which were additionally purified by µ-SPE were colorless and transparent. A comparison of chromatograms obtained from unpurified and µ-SPE purified blood samples (Figure 1) demonstrates the successful binding of analytes to the activated carbon and the elimination of all impurities, singling out only adenine nucleotides. The inclusion of an additional sample purification step is often required for biological samples to eliminate interfering peaks and to ensure uninterrupted HPLC system operation without frequent system washout. The applied µ-SPE method exhibited high reproducibility and precision, as evidenced by low RSD values between four blood samples: 11.4%, 10.9%, and 11.6% for ATP, ADP, and AMP, respectively. For comparison, RSD values between the four blood samples before the applied µ-SPE method were 7.4%, 8.4%, and 9.9% for ATP, ADP, and AMP. The mean values of AN loss after purification were determined to be 50.5% for ATP, 47.2% for ADP, and 41.7% for AMP, which were factored into the final concentration calculations after the µ-SPE method.

### 2.3. ATP, ADP, and AMP Chromatographic Separation and Quantification

**Figure 2:**
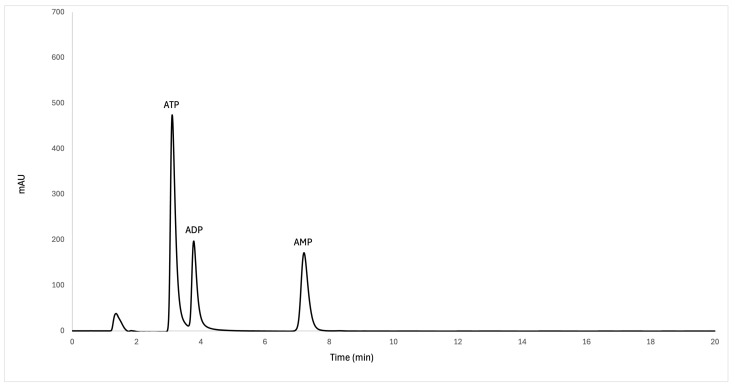
Magnified HPLC chromatogram of a 500 µM adenine nucleotide standard mix solution after HPLC method optimization. The x-axis represents retention time in minutes, and the y-axis measures a specific signal generated by detectors in milli-absorbance units (mAu).

Before selecting chromatographic conditions for method optimization, the properties of analytes should be investigated. Adenine nucleotides are considered polar organic anions [60,61], although it is interesting to mention that ATP exhibits hydrotropic properties due to the relatively hydrophobic aromatic ring of adenine and the highly polar triphosphate chain. The aromatic ring is able to cluster over hydrophobic regions of liquid droplets or aggregates, while the triphosphate chain interacts strongly with water molecules [62]. One of the goals of this research was to develop an efficient AN extraction protocol which would precede quantitative HPLC analysis of ANs in human blood samples. Several analytical methods have been developed for nucleotide analysis using either isocratic or gradient, ion-exchange or ion-pair (IP) reversed-phase (RP) HPLC in combination with UV or fluorescence detection [63]. In addition, an HPLC method based on hydrophilic interactions (HILICs) has been proposed as a newer, valid, and reliable alternative to the proposed methods [39,64]. Although good separation of purine nucleotides and nucleosides has been reported using ion-exchange HPLC [65,66], a major disadvantage of IEC is that the column packings are poorly reproducible and less stable, offering longer separation times than RP-HPLC [67]. RP columns offer higher efficiency and greater versatility compared to IEC columns [68,69]. However, since nucleotides are very polar compounds, they are retained to a lesser extent in conventional columns, especially when RP-HPLC is used. Therefore, the ion-pairing mode has been used to circumvent the poor retention of nucleotides in RP mode [68,69,70,71]. In general, ion-pair RP-HPLC has the advantages of both ion-exchange and RP methods, and numerous successful applications of ion-pair RP-HPLC have been reported for the analysis of adenine nucleotides using isocratic or gradient methods [14,60,63,72]. However, the mobile phases used in IP-RP-HPLC reduce the efficiency of the stationary phase for a shorter period of time than in the case of RP-HPLC [67]. Although many different packing materials can be used as stationary phases for RP-HPLC, one type is generally used for nucleotide analysis; that is, the octadecyl column [7,14,67,69,70,71]. For this reason, we used a Hypersil ODS filled with a silica gel coated with a monolayer of octadecylsilane in our study. In the first step, a previously published HPLC method was tested using an RP-HPLC method with a Hypersil ODS column for the quantification of ANs from human blood [7]. Slight adjustments in chromatography parameters were needed to account for the difference in column characteristics, a bigger particle size and shorter length compared to the column used by Coolen et al. [7]. Namely, the initial poor peak separation was significantly improved by decreasing the pH value of 50 mM phosphate buffer in mobile phase A to 6 instead of 7.7. Furthermore, setting the column temperature to 20 °C and reducing the flow rate to 0.6 mL/min significantly improved chromatographic separation of ATP and ADP peaks. Additionally, HPLC gradient elution was set to 30 min to adequately condition the chromatographic column prior to the next sample injection. Reducing the chromatography run time to 20 min led to the inconsistent chromatographic separation characterized by poor peak symmetry. The results of the standard solution mix analysis showed that the ATP molecule, which is the most polar, eluted first, at a retention time of 3.49 min, followed by ADP (4.17 min), and AMP (7.78 min) (Figure 2), which is to be expected for reverse phase chromatography.

Mean values of AN concentrations after extraction and purification were 1393.1 ± 189 µM for ATP, 254.8 ± 8 µM for ADP, and 76.9 ± 20 µM for AMP (Table 1). Notably, observed concentrations from blood samples aligned closely with previously reported values where the normal concentration of ATP in blood ranges from 1129.33 to 1386.50 µM, ADP values range from 168.74 to 288.02 µM, and AMP from 15.44 to 72.13 µM [6,8]. Intriguingly, although ATP-dependent enzymes require only micromolar concentrations of ATP as an energy source to drive chemical reactions [73], cellular concentrations of ATP are usually quite high, ranging from 1 to 12 mM, depending on cell types [62]. It has previously been reported by Patel et al. [73] that a ~50-fold higher AEC value is required to enable ATP-dependent metabolic reactions. Consequently, cells must maintain ATP in the millimolar range, but ADP and AMP at <50 μM and <1–10 μM, respectively [74,75], which is consistent with our results.

**Figure 3:**
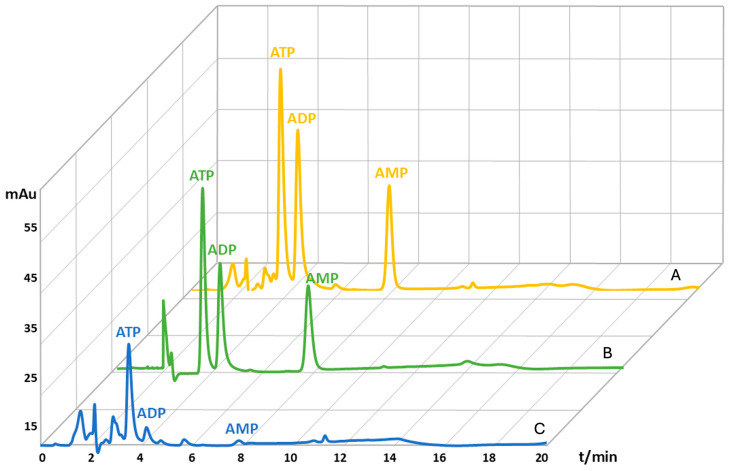
Magnified HPLC chromatograms of adenine nucleotides extracted from capillary blood spiked with a 200 µM standard solution mix (A), adenine nucleotides from a 200 µM standards solution mix (B), adenine nucleotides from capillary blood (C). The x-axis represents retention time in minutes, and the y-axis measures a specific signal generated by a detector in milli-absorbance units (mAu).

Subsequently, the AEC values for each blood sample were calculated according to the equation (Section 1), resulting in an AEC value of 0.88 ± 0.02 which is in accordance with the reported AEC values for healthy individuals that range between 0.8 and 0.9 [5]. Recovery was determined by comparing the values of spiked and non-spiked blood samples (Figure 3). The recovery value for the ATP molecule was 71.6%, for the ADP molecule was 121.8%, and for the AMP molecule 112.1% (Table 1). Recovery values higher than 100% for the ADP and AMP molecules indicate ATP hydrolysis to ADP and AMP [7,76,77,78,79,80]. The obtained values were used to correct the calculated concentrations of ATP, ADP, and AMP molecules from the blood samples. Moreover, blood samples stored at −80 °C for one week were stable as AN degradation was within 2% (results not shown).

### 2.4. HPLC Method Validation

Validation of the HPLC method was undertaken by evaluating linearity range, selectivity, inter-day and intra-day precision, limit of detection (LOD), limit of quantification (LOQ), recovery, and stability for each AN individually. The calculated coefficient of determination (*R*^2^) showed high linearity range for each AN (Supplementary Materials). Adequate selectivity was determined by observing the peak symmetry and resolution of each AN peak individually. Peak symmetries of the standard solution analysis were 0.51 for ATP, 0.53 for ADP, and 0.68 for AMP, respectively, indicating symmetrical peak shapes with minimal tailing or fronting [81]. In contrast, peak symmetries of ANs extracted from the blood sample were 0.58 for ATP, 0.64 for ADP, and 1.30 for AMP. Additionally, satisfactory repeatability of the method was determined by AN standard solution analysis in triplicates within the same day (intra-day) and between three consecutive days (inter-day). Relative standard deviation (RSD) for intra-day precision ranged from 0.01 to 0.86%, and from 0.01 to 1.22% for inter-day precision (Table 2). Low RSD values indicate a good precision and high reproducibility of the modified HPLC method. Furthermore, LOD values were 0.08 µM, 0.27 µM, and 0.15 µM for ATP, ADP, and AMP, respectively, and LOQ values were 0.27 µM, 0.91 µM, and 0.48 µM for ATP, ADP, and AMP, respectively. LOD and LOQ values were adequate for the objectives of this study.

## 3. Materials and Methods

### 3.1. Chemicals

Adenosine 5′-monophosphate (AMP), adenosine 5′-diphosphate (ADP), adenosine 5′-triphosphate (ATP) standard solutions, perchloric acid (PCA), formic acid, ammonium hydroxide, and methanol were purchased of analytical grade from Sigma-Aldrich (St. Louis, MO, USA). Sodium carbonate, sodium hydroxide, potassium hydrogen phosphate, and potassium dihydrogen phosphate were of analytical grade, purchased from Kemika (Zagreb, Croatia). Acetonitrile of HPLC grade was purchased from Merck Millipore (Burlington, MA, USA) and activated carbon used as a μ-SPE stationary phase was purchased from Harvard Apparatus (Holliston, MA, USA). Ultra-pure water was generated in-house (18.2 MΩ cm, Merck Millipore, Burlington, MA, USA) and was used in every step of the sample preparation.

### 3.2. Blood Sampling and Adenine Nucleotides Liquid-Liquid Extraction

**Figure 4:**
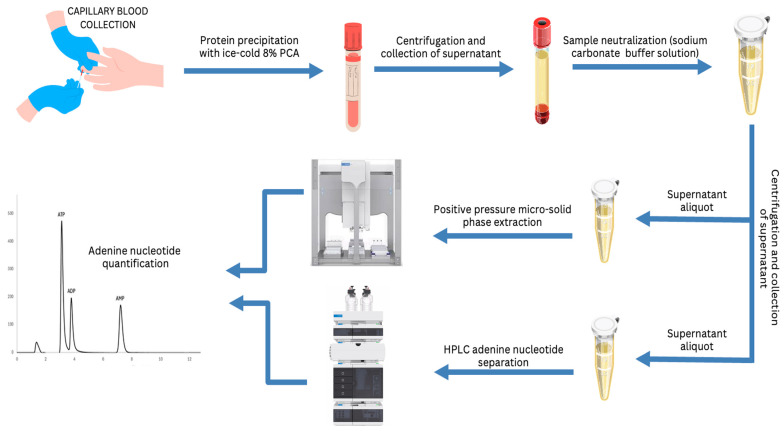
Schematic representation of the of extraction, purification, and quantification of adenine nucleotides from human blood using automated µ-SPE with positive pressure, and reverse-phase HPLC analysis.

The workflow of the whole protocol, starting from the blood collection to the adenine nucleotide detection and quantification is shown in Figure 4.

The AN extraction procedure was conducted on four capillary blood samples collected from a healthy female volunteer under the age of thirty, with a blood type B+ and normal hematological parameters, according to a modified method by Coolen et al. [7]. Capillary blood was collected by puncturing the index finger with a lancet, yielding a 50 µL blood sample. All the extraction steps were performed on ice. Immediately after sampling, 50 µL of capillary blood was mixed with an equal volume of ice-cold 8% (*v*/*v*) PCA to promote hemolysis and protein precipitation [82]. The obtained mixture was centrifuged at 16,000× *g* for 10 min at 4 °C (Microcentrifuge 5415, Eppendorf, Hamburg, Germany). A total of 65 µL of supernatant was transferred to a clean tube and neutralized with 4 µL of sodium carbonate buffer (2 M sodium carbonate in 6 M sodium hydroxide), followed by centrifugation at 16,000× *g* for 10 min at 4 °C. After that, 100 µL of visibly turbid supernatant was collected. To prevent HPLC clogging, the sample was purified using a Microcon-10 kDa filter (Merck Millipore, Burlington, MA, USA). An amount of 40 µL of the obtained filtrate was diluted with 160 µL of 50 mM phosphate buffer, pH 6, and divided into two aliquots. The first aliquot was immediately analyzed by reverse-phase (RP) HPLC, and the second one was subjected to µ-SPE purification prior the analysis.

### 3.3. Positive-Pressure µ-SPE Method

Method for the automated positive-pressure µ-SPE for sample purification was adapted from Pabst et al. [53] with modifications to suit our experimental requirements. The purification procedure was performed on the AssayMAP Bravo Platform (Agilent, St. Clara, CA, USA). Resin-free cartridges of 5 µL capacity were manually packed with activated carbon which was used as the stationary phase. Cartridges were primed with 300 µL of the priming buffer (acetonitrile in 3% formic acid, pH 9, 60:40; *v*/*v*) at a flow rate of 300 µL/min, followed by equilibration of the stationary phase with 100 µL of ultra-pure water (18.2 MΩ cm) and a flow rate of 100 µL/min. Subsequently, 100 µL of the sample was loaded onto the cartridge at a flow rate of 5 µL/min. An additional washing step with 50 µL of equilibration solution at a flow rate of 5 µL/min was included to remove unbound metabolites. Ultimately, ANs were eluted in 100 µL of the elution buffer (acetonitrile in 3% (*v*/*v*) formic acid, pH 9, 60:40) with 5 µL/min flow rate. Obtained eluates were vacuum dried (Eppendorf vacuum concentrator, Hamburg, Germany) and dissolved in 100 µL of 50 mM phosphate buffer (pH 6) for subsequent HPLC analysis. A summary of the entire µ-SPE protocol is shown in Table 3. After establishing a reproducible, accurate, and precise μ-SPE method using adenine nucleotide standard solutions, the study progressed to the analysis of multiple human blood samples.

### 3.4. Standard Solution Preparation

Standard solution mixtures of ATP, ADP, and AMP were used to optimize and validate the purification and HPLC detection methods. Each analyte (ATP, ADP, and AMP) was prepared as a stock solution in ice-cold 8% *(v*/*v)* PCA at a concentration of 1000 μM. These stock solutions were stored at −80 °C until use. Standard solution mixtures containing all three AN were prepared by diluting the stock solutions in ice-cold 8% *(v*/*v)* PCA to obtain concentrations of 10, 50, 100, 200, and 333.3 μM for each adenine nucleotide. Additionally, a blank sample consisting of 50 mM phosphate buffer was included to evaluate any potential interactions within the solution and to detect and exclude contaminations.

### 3.5. HPLC Analysis

The identification and quantification of individual ANs, both from standard solutions and extracted from blood, was achieved through RP-HPLC analysis. Chromatographic analyses were conducted on the Agilent 1100 Series HPLC system (St. Clara, CA, USA) equipped with a diode-array detector (DAD), a Hypersil ODS C18 column with a particle size of 5 µm (125 mm × 4 mm; Waltham, MA, USA), and a Hypersil ODS (10 mm × 4 mm, 5 µm) guard column. The sample injection volume was set to 25 µL, and the column temperature was maintained at 20 °C throughout the analyses. The total duration of each chromatographic run was 30 min at a constant flow rate of 0.6 mL/min. ANs were identified by their respective retention times, which were derived from standard solutions, and the detection wavelength was set to 260 nm. The mobile phase was composed of 50 mM phosphate buffer, pH 6 (mobile phase A), and 100% methanol (mobile phase B). Gradient elution was performed by increasing the percentage of mobile phase B as presented in Table 4. Peak integration and data analysis were performed using Agilent ChemStation software B.04.03 SP1 (St. Clara, CA, USA).

### 3.6. HPLC Method Validation

The presented HPLC method vas validated with respect to linearity range, selectivity, inter-day and intra-day precision, limit of detection (LOD), limit of quantification (LOQ), recovery, and stability for each tested AN. Linearity was evaluated by analyzing five concentrations of AN standard solutions (10 µM, 50 µM, 100 µM, 200 µM, 333.3 µM) in triplicates. Linearity was checked by plotting calibration curves of the average peak area of the individual AN against the corresponding concentration. Slope, correlation coefficient, and intercept were determined using linear least squares analysis. Chromatographic selectivity was determined by observing the resolution, separation, and symmetry of AN peaks derived from the analysis of the standard solutions. Precision was assessed by calculating the relative standard deviation (RSD) of standard solutions analyzed in triplicates over a single day (intra-day precision) and across three consecutive days (inter-day precision). LOD and LOQ were determined from a constructed calibration curve for each standard solution concentration using the following equations:

(2) $$LOD=3.3\times \frac{Sxy}{\alpha }$$

(3) $$LOQ=10\times \frac{Sxy}{\alpha }$$

where Sxy represents the standard error of the regression and α denotes the slope of the calibration curve. The method was also validated with regard to the recovery and stability of the sample. Sample stability was evaluated by comparing the AN concentrations immediately after extraction with those after one week of storage at −80 °C. In Supplement Table S1, a table has been compiled to highlight methodological advancements in this research, compared to existing studies. The table summarizes key parameters from related works.

The standard addition method involves adding known amounts of analyte to an unknown sample, a process known as spiking. By increasing the number of spikes, the analyst can extrapolate for the analyte concentration in the unknown that has not been spiked [83]. The standard addition method was used to calculate recoveries of each analyte by spiking capillary blood samples with 8% (*v*/*v*) PCA containing a known concentration (200 µM) of each adenine nucleotide. Recovery was calculated using the following equation:

(4) $$Recovery\left(\%\right)=\frac{\left[(spiked\;analyte\;concentration-unspiked\;analyte\;concetration)\right]}{spike\;concentration}\times 100$$

## 4. Conclusions

The presented AN extraction and quantification workflow proved to have high sensitivity as it requires only a drop of capillary human blood, which, according to the literature, is the smallest amount of blood used for AN analysis. The µ-SPE with activated carbon as the stationary phase represents a purification step that has proven to be efficient in impurities removal, and therefore beneficial for the subsequent HPLC detection and quantification of the AN. More specifically, the µ-SPE procedure enabled subsequent higher signal intensity and better chromatographic separation of ATP, ADP, and AMP, compared to the sample not additionally purified by SPE. The higher peak selectivity and resolution led to easier and more accurate quantification, compared to the blood samples that were not processed prior to analysis. Finally, the developed workflow enables the processing of a large number of blood samples in a short time, while maintaining accuracy, precision, and reproducibility. The established methodological framework is minimally invasive and the small sample volume is practical for AN analysis of various biological samples, such as cell cultures, follicular fluid, seminal plasma, placenta, and others. AN molecules are essential for all metabolic processes in the human body; therefore, determining their concentration in human blood has potential as a useful biomarker for many metabolic disorders and pathological conditions.

## Figures and Tables

**Table 1:** Recovery data of adenine nucleotides extracted from human capillary blood. The concentrations presented are mean values of *N* = 4 (blood samples), *N* = 4 (blood + spike samples).

Adenine Nucleotide	Blood (µM)	Spike (µM)	Blood + Spike (µM)	Recovery (%)
ATP	1393.1	1983.4	2807.9	71.6
ADP	254.8	1618.8	2228.2	121.8
AMP	76.9	1332.2	1573.8	112.1

**Table 2:** Intra-day and inter-day accuracy for ATP, ADP, and AMP standard solutions in the concentrations of 10, 50, 100, 200, and 333.3 µM.

Adenine Nucleotide	Theoretical Concentration (µM)	Intra-Day		Inter-Day	
		Observed Concentration (µM)	RSD (%)	Observed Concentration (µM)	RSD (%)
ATP	10	9.47 ± 0.75	0.24	9.16 ± 0.21	0.07
	50	50.27 ± 3.35	0.22	49.97 ± 2.80	0.19
	100	100.22 ± 4.16	0.14	98.43 ± 2.25	0.08
	200	199.9 ± 2.12	0.03	201.43 ± 1.40	0.02
	333.3	332.21 ± 1.15	0.01	331.74 ± 3.13	0.03
ADP	10	9.61 ± 2.51	0.86	9.22 ± 0.92	0.32
	50	50.91 ± 3.16	0.22	49.22 ± 1.70	0.12
	100	99.14 ± 2.60	0.09	100.15 ± 0.71	0.03
	200	198.24 ± 1.53	0.03	200.00 ± 5.37	0.10
	333.3	332.52 ± 1.10	0.01	331.87 ± 3.68	0.04
AMP	10	10.23 ± 1.30	0.46	10.37 ± 3.48	1.22
	50	50.69 ± 1.82	0.13	49.47 ± 4.36	0.33
	100	99.46 ± 0.91	0.03	99.87 ± 0.50	0.02
	200	198.81 ± 2.76	0.05	198.14 ± 0.62	0.01
	333.3	333.71 ± 1.81	0.02	331.20 ± 1.25	0.01

**Table 3:** The summary of µ-SPE protocol detailing the used solutions, volumes, and flow rates for each step of the procedure.

µ-SPE Step	Solution	Volume (µL)	Flow Rate (µL/min)
Priming	Acetonitrile in 3% formic acid, pH 9, 60:40; *v*/*v*	300	100
Equilibration	ultra-pure water	100	100
Sample load	Standard solution and capillary blood	100	5
Washing	Ultra-pure water	100	5
Elution	Acetonitrile in 3% formic acid, pH 9, 60:40; *v*/*v*	25	5

**Table 4:** Elution gradient composition used for RP-HPLC method.

Time (min)	Flow Rate (mL/min)	Mobile Phase Solution A 50 mM Phosphate Buffer (%)	Mobile Phase Solution B 100% Methanol (%)
0.0	0.6	100.0	0.0
2.0	0.6	100.0	0.0
10.0	0.6	87.5	12.5
12.0	0.6	87.5	12.5
20.0	0.6	100.0	0.0
30.0	0.6	100.0	0.0

## Data Availability

The data presented in this study are available in the article and Supplementary Materials.

## Supplementary Materials

The following supporting information can be downloaded at https://www.mdpi.com/article/10.3390/molecules29235630/s1, Figure S1: Standard curve for ATP molecule quantification made from ATP standard solution in the concentrations of 10, 50, 100, 200, and 333.3 µM; Figure S2: Standard curve for ADP molecule quantification made from ADP standard solution in the concentrations of 10, 50, 100, 200, and 333.3 µM; Figure S3: Standard curve for AMP molecule quantification made from AMP standard solution in the concentrations of 10, 50, 100, 200, and 333.3 µM; Table S1: Comparison of methodology in similar research.

## References

[1] Burnstock G. (2013). Purinergic signalling: Pathophysiology and therapeutic potential. Keio J. Med..

[2] Aragon-Martinez O.H., Galicia O., Isiordia-Espinoza M.A., Martinez-Morales F. (2014). A novel method for measuring the ATP-related compounds in human erythrocytes. Tohoku J. Exp. Med..

[3] Atkinson D.E. (1968). The energy charge of the adenylate pool as a regulatory parameter. Interaction with feedback modifiers. Biochemistry.

[4] Oakhill J.S., Steel R., Chen Z.P., Scott J.W., Ling N., Tam S. (2011). AMPK is a direct adenylate charge-regulated protein kinase. Science.

[5] Zhang L., Vertes A. (2015). Energy Charge, Redox State, and Metabolite Turnover in Single Human Hepatocytes Revealed by Capillary Microsampling Mass Spectrometry. Anal. Chem..

[6] la Fuente I.M.D., Cortes J.M., Valero E., Desroches M., Rodrigues S., Martinez L. (2014). On the Dynamics of the Adenylate Energy System: Homeorhesis vs Homeostasis. PLoS ONE.

[7] Coolen E.J.C.M., Arts I.C.W., Swennen E.L.R., Bast A., Stuart M.A.C., Dagnelie P.C. (2008). Simultaneous determination of adenosine triphosphate and its metabolites in human whole blood by RP-HPLC and UV-detection. J. Chromatogr. B Analyt. Technol. Biomed. Life. Sci..

[8] Domański L., Safranow K., Ostrowski M., Pawlik A., Olszewska M., Dutkiewicz G., Ciechanowski K. (2007). Oxypurine and purine nucleoside concentrations in renal vein of allograft are potential markers of energy status of renal tissue. Arch. Med. Res..

[9] Marlewski M., Smolenski R.T., Szolkiewicz M., Aleksandrowicz Z., Rutkowski B., Swierczynski J. (2000). Accelerated degradation of adenine nucleotide in erythrocytes of patients with chronic renal failure. Mol. Cell. Biochem..

[10] Lichtman M.A., Miller D.R. (1970). Erythrocyte glycolysis, 2,3-diphosphoglycerate and adenosine triphosphate concentration in uremic subjects: Relationship to extracellular phosphate concentration. J. Lab. Clin. Med..

[11] Wallas C.H. (1974). Metabolic studies on red cells from patients with chronic renal disease on haemodialysis. Br. J. Haematol..

[12] Zhang C., Liu Z., Liu X., Wei L., Liu Y., Yu J., Sun L. (2013). Targeted metabolic analysis of nucleotides and identification of biomarkers associated with cancer in cultured cell models. Acta Pharm. Sin. B.

[13] Ledderose C., Valsami E.A., Newhams M., Novak T., Randolph A.G., Junger W.G. (2023). ATP breakdown in plasma of children limits the antimicrobial effectiveness of their neutrophils. Purinergic Signal..

[14] Redžović Z., Erk M., Svetličić E., Dončević L., Gottstein S., Hozić A., Cindrić M.M. (2021). Determination of adenylate nucleotides in amphipod *Gammarus fossarum* by ion-pair reverse phase liquid chromatography: Possibilities of positive pressure micro-solid phase extraction. Separations.

[15] Telesiński A. (2010). The effects of 2, 4-D and dicamba on isoproturon metabolism and selected biochemical parameters in clay soil. Electron. J. Pol. Agric. Univ..

[16] Popova A., Kemp R.B. (2002). The adenylate energy charge in marine microplankton under different levels of pollution by oil products and the stages of seasonal succession. Int. J. Algae.

[17] Koop J.H.E., Winkelmann C., Becker J., Hellmann C., Ortmann C. (2011). Physiological indicators of fitness in benthic invertebrates: A useful measure for ecological health assessment and experimental ecology. Aquat. Ecol..

[18] Matoo O.B., Lannig G., Bock C., Sokolova I.M. (2021). Temperature but not ocean acidification affects energy metabolism and enzyme activities in the blue mussel, Mytilus edulis. Ecol. Evol..

[19] Ledderose C., Valsami E.A., Wolfgang W.J. (2022). Optimized HPLC method to elucidate the complex purinergic signaling dynamics that regulate ATP, ADP, AMP, and adenosine levels in human blood. Purinergic Signal..

[20] Yeung P., Ding L., Casley W.L. (2008). HPLC assay with UV detection for determination of RBC purine nucleotide concentrations and application for biomarker study in vivo. J. Pharm. Biomed. Anal..

[21] Baranowska-Bosiacka I., Hlynczak A.J. (2004). Effect of lead ions on rat erythrocyte purine content. Biol. Trace Elem. Res..

[22] Dudzinska W., Hlynczak A.J. (2004). Purine nucleotides and their metabolites in erythrocytes of streptozotocin diabetic rats. Diabetes Metab..

[23] Smoleńska Z., Kaznowska Z., Zarówny D., Simmonds H.A., Smoleński R.T. (1999). Effect of methotrexate on blood purine and pyrimidine levels in patients with rheumatoid arthritis. Rheumatology.

[24] Dudzinska W., Lubkowska A., Dolegowska B., Safranow K., Jakubowska K. (2010). Adenine, guanine and pyridine nucleotides in blood during physical exercise and restitution in healthy subjects. Eur. J. Appl. Physiol..

[25] Zamaraeva M., Sabirov R., Maeno E., Ando-Akatsuka Y., Bessonova S., Okada Y. (2005). Cells die with increased cytosolic ATP during apoptosis: A bioluminescence study with intracellular luciferase. Cell Death Differ..

[26] Patergnani S., Baldassari F., De Marchi E., Karkucinska-Wieckowska A., Wieckowski M.R., Pinton P. (2014). Chapter Sixteen—Methods to Monitor and Compare Mitochondrial and Glycolytic ATP Production. Methods in Enzymology.

[27] Contreras-Sanz A., Scott-Ward T.S., Gill H.S., Jacoby J.C., Birch R.E., Malone-Lee J., Taylor K.M.G., Peppiatt-Wildman C.M., Wildman S.S.P. (2012). Simultaneous quantification of 12 different nucleotides and nucleosides released from renal epithelium and in human urine samples using ion-pair reversed-phase HPLC. Purinergic Signal..

[28] Straube H., Witte C.-P., Herde M. (2021). Analysis of Nucleosides and Nucleotides in Plants: An Update on Sample Preparation and LC-MS Techniques. Cells.

[29] Ullrich J., Calvin M. (1962). Alcohol-resistant phosphatase activity in chloroplasts. Biochim. Biophys. Acta.

[30] Ullrich J. (1963). Phosphatase action on phosphoglycolic, 3-phosphoglyceric, and phosphoenol pyruvic acids in spinach chloroplast fragments in the presence and absence of high concentrations of methanol. Biochim. Biophys. Acta.

[31] Ikuma H., Tetley R.M. (1976). Possible Interference by an Acid-stable Enzyme during the Extraction of Nucleoside Di- and Triphosphates from Higher Plant Tissues. Plant Physiol..

[32] Straube H., Niehaus M., Zwittian S., Witte C.-P., Herde M. (2021). Enhanced nucleotide analysis enables the quantification of deoxynucleotides in plants and algae revealing connections between nucleoside and deoxynucleoside metabolism. Plant Cell.

[33] Bieleski R.L. (1964). The problem of halting enzyme action when extracting plant tissues. Anal. Biochem..

[34] Khym J.X. (1975). An Analytical System for Rapid Separation of Tissue Nucleotides at Low Pressures on Conventional Anion Exchangers. Clin. Chem..

[35] Naing N.N., Tan S.C., Lee H.K., Poole C. (2019). Micro-solid-phase extraction. Solid-Phase Extraction.

[36] Czarnecka J., Cieślak M., Michał K. (2005). Application of solid phase extraction and high-performance liquid chromatography to qualitative and quantitative analysis of nucleotides and nucleosides in human cerebrospinal fluid. J. Chromatogr. B.

[37] Svensson J.O., Jonzon B. (1990). Determination of adenosine and cyclic adenosine monophosphate in urine using solid-phase extraction and high-performance liquid chromatography with fluorimetric detection. J. Chromatogr. B Biomed. Sci. Appl..

[38] Guérard F., Pétriacq P., Gakière B., Tcherkez G. (2011). Liquid chromatography/time-of-flight mass spectrometry for the analysis of plant samples: A method for simultaneous screening of common cofactors or nucleotides and application to an engineered plant line. Plant Physiol. Biochem..

[39] Guo M., Yin D., Han J., Zhang L., Li X., Tang D. (2016). Phenylboronic acid modified solid-phase extraction column: Preparation, characterization, and application to the analysis of amino acids in sepia capsule by removing the maltose. J. Sep. Sci..

[40] Strezsak S.R., Beuning P.J., Skizim N.J. (2022). Versatile separation of nucleotides from bacterial cell lysates using strong anion exchange chromatography. J. Chromatogr. B.

[41] Janas E., Hofacker M., Chen M., Gompf S., van der Does C., Tampé R. (2003). The ATP Hydrolysis Cycle of the Nucleotide-binding Domain of the Mitochondrial ATP-binding Cassette Transporter Mdl1p*. J. Biol. Chem..

[42] Bates-Fraser L.C., Moertl K.M., Stopforth C.K., Bartlett D.B., Ondrak K.S., Jensen B.C., Hanson E.D. (2024). A practical approach for complete blood count analysis following acute exercise: Capillary vs. venous blood sampling. Adv. Exerc. Health Sci..

[43] Souverain S., Rudaz S., Veuthey J.-L. (2004). Protein precipitation for the analysis of a drug cocktail in plasma by LC–ESI–MS. J. Pharm. Biomed. Anal..

[44] Stone J., Nair H., Clarke W. (2017). Chapter 3—Sample preparation techniques for mass spectrometry in the clinical laboratory. Mass Spectrometry for the Clinical Laboratory.

[45] Lim C.K. (1988). Sample preparation for high-performance liquid chromatography in the clinical laboratory. TrAC Trends Anal. Chem..

[46] Jemal M. (2000). High-throughput quantitative bioanalysis by LC/MS/MS. Biomed. Chromatogr. BMC.

[47] McDowall R.D. (1989). Sample preparation for biomedical analysis. J. Chromatogr..

[48] Makszin L., Kustan P., Szirmay B., Pager C., Mezo E., Kalacs K., Paszthy V., Gyorgy E., Kilar F., Ludany A. (2018). Microchip gel electrophoretic analysis of perchloric acid-soluble serum proteins in systemic inflammatory disorders. Electrophoresis.

[49] PCA Deproteinization Protocol | Abcam. [Online]. https://www.abcam.com/en-us/technical-resources/protocols/deproteinization.

[50] Williams C., Forrester T. (1976). Loss of ATP in micromolar amounts after perchloric acid treatment. Pflug. Arch..

[51] Thorfinnsdottir L.B., García-Calvo L., Bø G.H., Bruheim P., Røst L.M. (2023). Optimized Fast Filtration-Based Sampling and Extraction Enables Precise and Absolute Quantification of the Escherichia coli Central Carbon Metabolome. Metabolites.

[52] Bladergroen M.R., van der Burgt Y.E.M. (2015). Solid-Phase Extraction Strategies to Surmount Body Fluid Sample Complexity in High-Throughput Mass Spectrometry-Based Proteomics. J. Anal. Methods Chem..

[53] Pabst M., Grass J., Fischl R., Léonard R., Jin C., Hinterkörner G. (2010). Nucleotide and nucleotide sugar analysis by liquid chromatography—Electrospray ionization-mass spectrometry on surface-conditioned porous graphitic carbon. Anal. Chem..

[54] Kong Z., Jia S., Chabes A.L., Appelblad P., Lundmark R., Moritz T., Chabes A. (2018). Simultaneous determination of ribonucleoside and deoxyribonucleoside triphosphates in biological samples by hydrophilic interaction liquid chromatography coupled with tandem mass spectrometry. Nucleic Acids Res..

[55] Bushman L.R., Kiser J.J., Rower J.E., Klein B., Zheng J.H., Ray M.L., Anderson P.L. (2011). Determination of nucleoside analog mono-, di-, and tri-phosphates in cellular matrix by solid phase extraction and ultra-sensitive LC-MS/MS detection. J. Pharm. Biomed. Anal..

[56] Mu L., Liu X., Li S., Tang F., Yu P. (2014). Determination of Intracellular Concentrations of Nucleoside Analogues and their Phosphorylated Metabolites. J. Mol. Pharm. Org. Process Res..

[57] Robbins B.L., Poston P.A., Neal E.F., Slaughter C., Rodman J.H. (2007). Simultaneous measurement of intracellular triphosphate metabolites of zidovudine, lamivudine and abacavir (carbovir) in human peripheral blood mononuclear cells by combined anion exchange solid phase extraction and LC-MS/MS. J. Chromatogr. B Analyt. Technol. Biomed. Life. Sci..

[58] Emotte C., Deglave F., Heudi O., Picard F., Kretz O. (2012). Fast simultaneous quantitative analysis of FTY720 and its metabolite FTY720-P in human blood by on-line solid phase extraction coupled with liquid chromatography–tandem mass spectrometry. J. Pharm. Biomed. Anal..

[59] Magdenoska O., Martinussen J., Thykaer J., Nielsen K.F. (2013). Dispersive solid phase extraction combined with ion-pair ultra high-performance liquid chromatography tandem mass spectrometry for quantification of nucleotides in Lactococcus lactis. Anal. Biochem..

[60] Uesugi T., Sano K., Uesawa Y., Ikegami Y., Mohri K. (1997). Ion-pair reversed-phase high-performance liquid chromatography of adenine nucleotides and nucleoside using triethylamine as a counterion. J. Chromatogr. B Biomed. Sci. Appl..

[61] Zhang G., Walker A.D., Lin Z., Han X., Blatnik M., Steenwyk R.C., Groeber E.A. (2014). Strategies for quantitation of endogenous adenine nucleotides in human plasma using novel ion-pair hydrophilic interaction chromatography coupled with tandem mass spectrometry. J. Chromatogr. A.

[62] Kang J., Lim L., Song J. (2019). ATP binds and inhibits the neurodegeneration-associated fibrillization of the FUS RRM domain. Commun. Biol..

[63] Bhatt D.P., Chen X., Geiger J.D., Rosenberger T.A. (2012). A sensitive HPLC-based method to quantify adenine nucleotides in primary astrocyte cell cultures. J. Chromatogr. B.

[64] Mora L., Hernández-Cázares A.S., Aristoy M.-C., Toldrá F. (2010). Hydrophilic interaction chromatographic determination of adenosine triphosphate and its metabolites. Food Chem..

[65] Hsu D.-S., Chen S.S. (1980). Simple anion-exchange chromatography for the determination of adenine nucleotides by using AG MP-1 resin. J. Chromatogr. A.

[66] Bartolini M., Wainer I.W., Bertucci C., Andrisano V. (2013). The rapid and direct determination of ATPase activity by ion exchange chromatography and the application to the activity of heat shock protein-90. J. Pharm. Biomed. Anal..

[67] Studzińska S., Buszewski B. (2013). Effect of mobile phase pH on the retention of nucleotides on different stationary phases for high-performance liquid chromatography. Anal. Bioanal. Chem..

[68] Qian T., Cai Z., Yang M.S. (2004). Determination of adenosine nucleotides in cultured cells by ion-pairing liquid chromatography–electrospray ionization mass spectrometry. Anal. Biochem..

[69] Cichna M., Raab M., Daxecker H., Griesmacher A., Müller M.M., Markl P. (2003). Determination of fifteen nucleotides in cultured human mononuclear blood and umbilical vein endothelial cells by solvent generated ion-pair chromatography. J. Chromatogr. B.

[70] Ganzera M., Vrabl P., Wörle E., Burgstaller W., Stuppner H. (2006). Determination of adenine and pyridine nucleotides in glucose-limited chemostat cultures of Penicillium simplicissimum by one-step ethanol extraction and ion-pairing liquid chromatography. Anal. Biochem..

[71] Bolin C., Cardozo-Pelaez F. (2007). Assessing biomarkers of oxidative stress: Analysis of guanosine and oxidized guanosine nucleotide triphosphates by high performance liquid chromatography with electrochemical detection. J. Chromatogr. B.

[72] Nedden S.Z., Eason R., Doney A.S., Frenguelli B.G. (2009). An ion-pair reversed-phase HPLC method for determination of fresh tissue adenine nucleotides avoiding freeze–thaw degradation of ATP. Anal. Biochem..

[73] Patel A., Malinovska L., Saha S., Wang J., Alberti S., Krishnan Y., Hyman A.A. (2017). ATP as a biological hydrotrope. Science.

[74] Cox M., Nelson D. (2000). Lehninger Principles of Biochemistry.

[75] Rice A.M., Rosen M.K. (2017). ATP controls the crowd. Science.

[76] Harmsen E., de Jong J.W., Serruys P.W. (1981). Hypoxanthine production by ischemic heart demonstrated by high pressure liquid chromatography of blood purine nucleosides and oxypurines. Clin. Chim. Acta Int. J. Clin. Chem..

[77] Crescentini G., Stocchi V. (1984). Fast reversed-phase high-performance liquid chromatographic determination of nucleotides in red blood cells. J. Chromatogr. A.

[78] Stocchi V., Cucchiarini L., Canestrari F., Piacentini M.P., Fornaini G. (1987). A very fast ion-pair reversed-phase HPLC method for the separation of the most significant nucleotides and their degradation products in human red blood cells. Anal. Biochem..

[79] Tekkanat K.K., Fox I.H. (1988). Isocratic separation of ATP and its degradation products from biological fluids by automated liquid chromatography. Clin. Chem..

[80] Caruso R., Campolo J., Dellanoce C., Mariele R., Parodi O., Accinni R. (2004). Critical study of preanalytical and analytical phases of adenine and pyridine nucleotide assay in human whole blood. Anal. Biochem..

[81] Wahab M.F., Patel D.C., Armstrong D.W. (2017). Total peak shape analysis: Detection and quantitation of concurrent fronting, tailing, and their effect on asymmetry measurements. J. Chromatogr. A.

[82] Ivanov I.T. (1999). Low pH-induced hemolysis of erythrocytes is related to the entry of the acid into cytosole and oxidative stress on cellular membranes. Biochim. Biophys. Acta.

[83] Andersen J.E.T. (2017). The standard addition method revisited. TrAC Trends Anal. Chem..

