# Supplementary material for: Advancements in Adenine Nucleotides Extraction and Quantification from a Single Drop of Human Blood
Source: Molecules. 2024 Nov 28;29(23):5630. doi: 10.3390/molecules29235630 (PMC11643363; doi:10.3390/molecules29235630)
Supplement: Supplementary file 1 [file molecules-29-05630-s001.zip › Table S1.pdf]

	Sample type	SPE method	AN detection method	LC column	Analysis time	LOD/LOQ
Coolen et al. 2008	Venous blood	None	HPLC	Hypersil ODS C18 RP column, 3 m particle size (150 mm × 4.6 mm), and a 5 m Hypersil C18 (10 mm × 4 mm) guard column	19 min	LOD values were below 0.15 μM<br><br>LOQ values were below 0.5 μM
Aragon-martinez et al. 2010	Venous blood	None	HPLC	Zorbax SB-C18 column, 5-μm particle size (250 mm × 4.6 mm)	20 min	LOD values were below 0.1 μM<br><br>LOQ values were below 0.3 μM
Pabst et al. 2010	Chinese hamster ovary cells, plant tissue and mouse liver	Hypercarb	LC-MS	Hypercarb PGC columns (0.32 × 100 mm)	26 min	LOD dictated by the Q-TOF's detector were 25 pmol on column.
Popović et al. 2024	Capillary blood	Activated carbon	HPLC	Hypersil ODS C18 column, 5 μm particle size (125 mm x 4 mm), and a Hypersil ODS (10 mm x 4 mm, 5 μm) guard column	30 min	LOD values were below 0.27 μM,<br><br>LOQ values were below 0.91 μM,
