# Supplementary figures and images for: Advancements in Adenine Nucleotides Extraction and Quantification from a Single Drop of Human Blood
Source: Molecules. 2024 Nov 28;29(23):5630. doi: 10.3390/molecules29235630 (PMC11643363; doi:10.3390/molecules29235630)

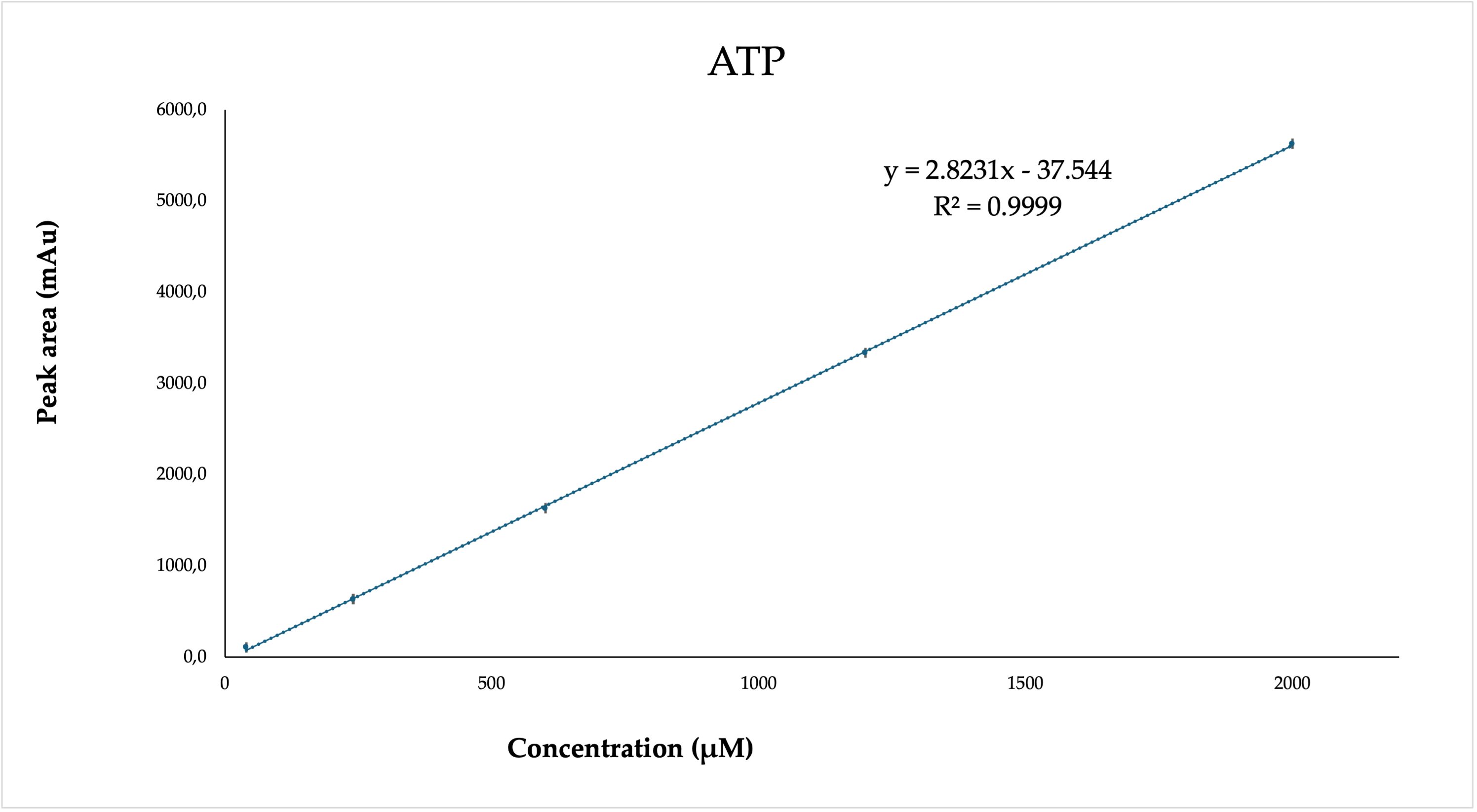

Supplement: Supplementary file 1 [file molecules-29-05630-s001.zip › Figure S1.png]

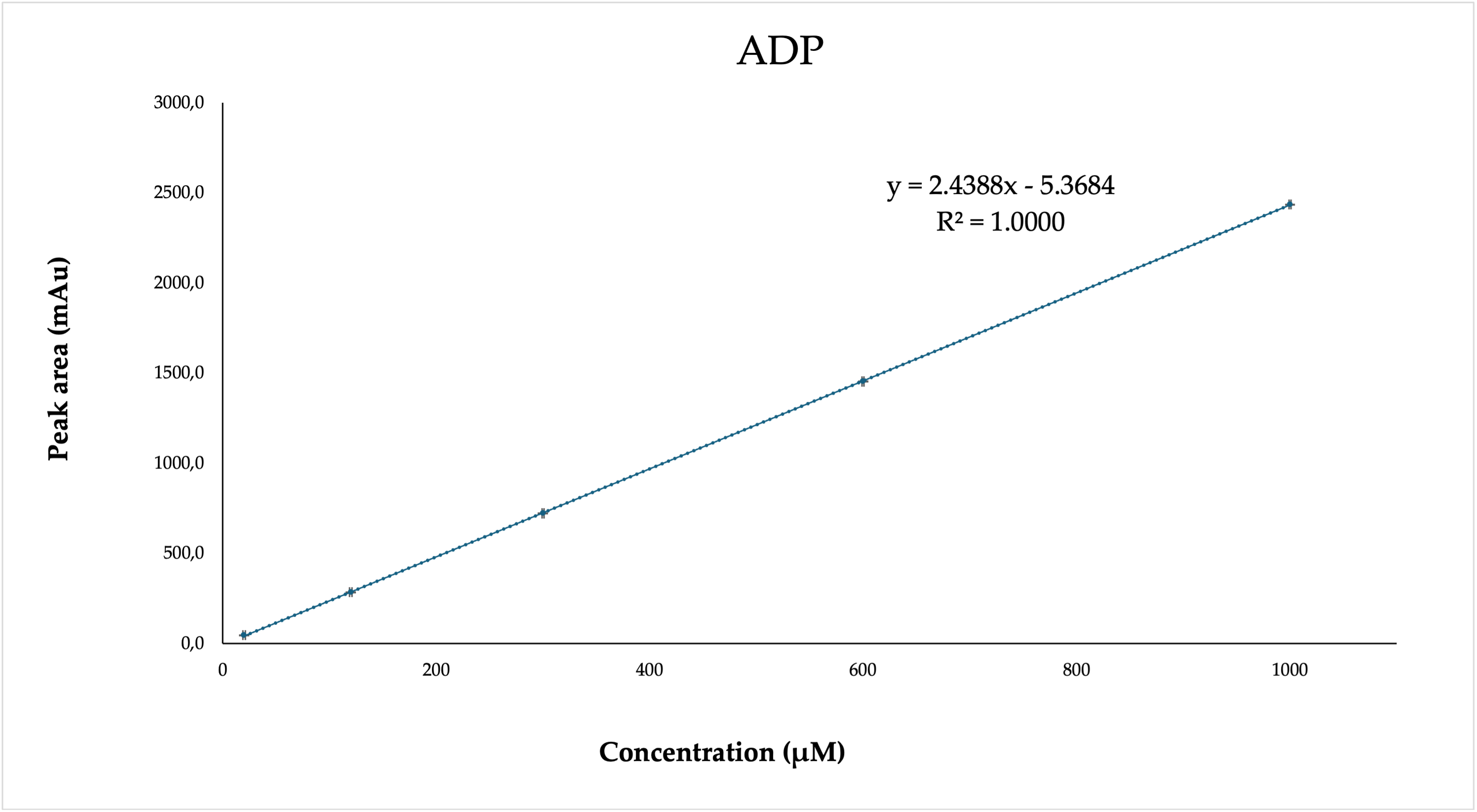

Supplement: Supplementary file 1 [file molecules-29-05630-s001.zip › Figure S2.png]

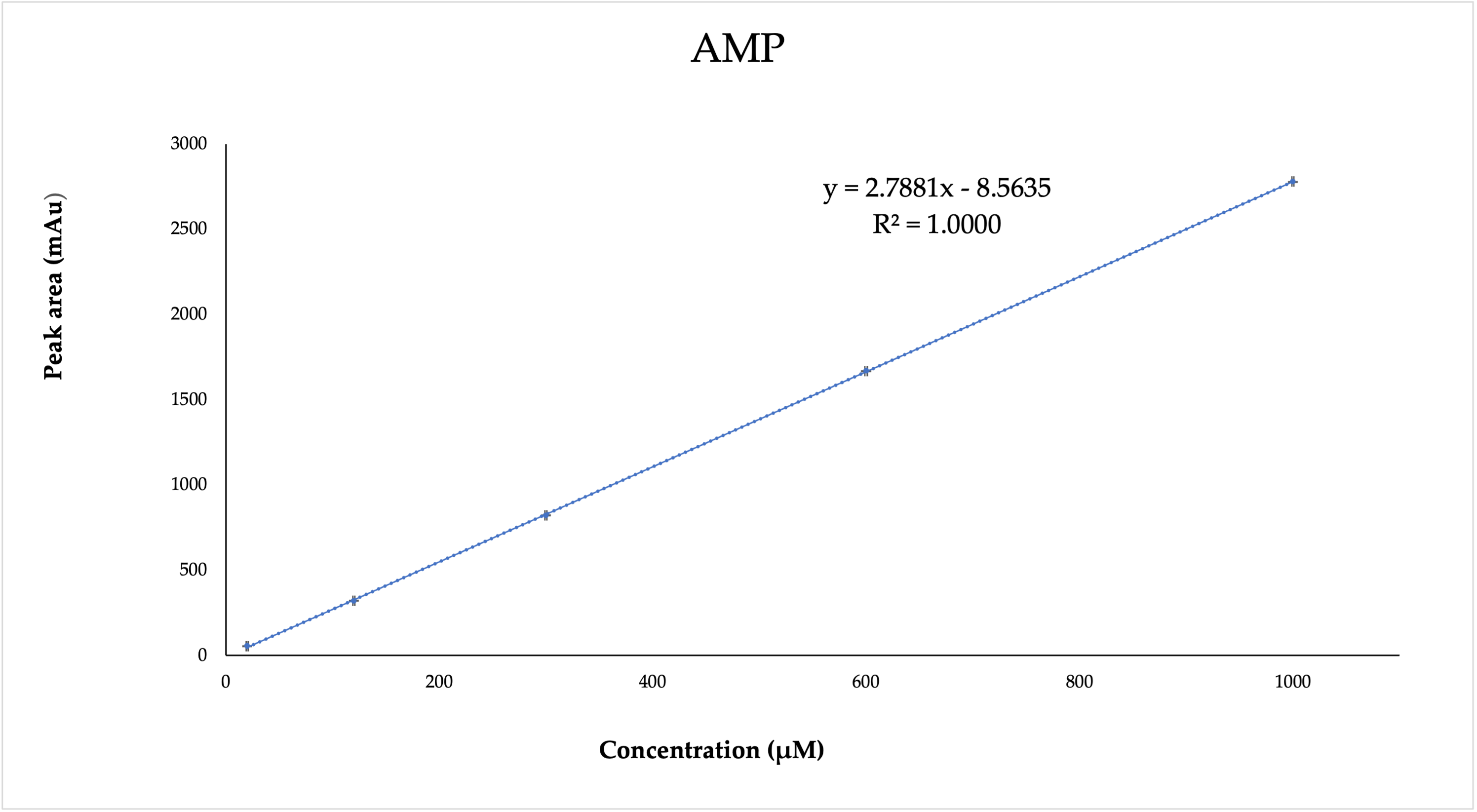

Supplement: Supplementary file 1 [file molecules-29-05630-s001.zip › Figure S3.png]
